# Prevalence, Awareness, and Control of Hypertension in Greater Beirut Area, Lebanon

**DOI:** 10.1155/2018/5419861

**Published:** 2018-12-26

**Authors:** Aya Noubani, Lara Nasreddine, Abla Mehio Sibai, Hani Tamim, Hussain Isma'eel

**Affiliations:** ^1^American University of Beirut, Faculty of Health Sciences, Department of Epidemiology and Population Health, Beirut, Lebanon; ^2^American University of Beirut Medical Center, Vascular Medicine Program, Beirut, Lebanon; ^3^American University of Beirut, Faculty of Agricultural and Food Sciences, Department of Nutrition and Food Sciences, Beirut, Lebanon; ^4^American University of Beirut Medical Center, Faculty of Medicine, Clinical Research Institute, Beirut, Lebanon; ^5^American University of Beirut Medical Center, Faculty of Medicine, Department of Internal Medicine, Beirut, Lebanon; ^6^American University of Beirut, Faculty of Medicine, Department of Internal Medicine, Division of Cardiology, Beirut, Lebanon

## Abstract

**Background:**

Hypertension (HTN) has been identified as the leading risk factor for mortality and the third cause of disability worldwide. Lebanon has witnessed a threefold increase in the prevalence of HTN in the past decade. The timely exploration and detection of the factors contributing to a higher prevalence of the disease among the Lebanese population is fundamental. The objectives of this study were to assess the prevalence, awareness, and control rates of HTN in Greater Beirut Area in Lebanon and to identify their respective predictors.

**Methods:**

A representative sample of 501 participants aged 18-79 years residing in Greater Beirut Area was examined. Data collection form was filled up, through interviews, physical exams, and lab tests. The analysis was done for three defined outcomes: blood pressure status (normotensive, prehypertension, and hypertension), unaware HTN, and uncontrolled HTN. These were compared for the various associated predictors.

**Results:**

The sample consisted of 64.3% women and mean age 45.4 ± 15 years and the subjects were predominantly from low educational income levels. The results showed that 36.4% of the study participants were hypertensive, 25.3% were prehypertensive, and 38.2% had optimal blood pressure, while the awareness rate was 65.4% and control rate was 61%. The independent predictors of HTN were age, gender, marital status, T2D, body fat, triglyceride (positive correlates), and income level (negative correlate). Moreover, unawareness of HTN was common among older age, men, single participants, and the obese. We could not identify any factor related to uncontrolled HTN.

**Conclusion:**

The trend in the prevalence of HTN in Greater Beirut Area is found to be consistent and relatively high, yet there was an observed improvement in the awareness and control of the disease. Public health measures on a national level are urgently needed to curb the increasing prevalence of HTN, achieve primary prevention, and better control the disease.

## 1. Introduction

HTN has been identified as the leading risk factor for mortality and is ranked as the third cause of disability worldwide [1]. Globally, it has been estimated that 9.4 million deaths annually are due to complications of HTN, such that 45% of deaths are due to heart diseases and 51% of deaths are due to stroke [2]. Therefore, HTN is a driving factor of the global burden of cardiovascular diseases and its complications. Moreover, this is expected to increase as projections estimate that there will be a 30% increase in the prevalence of HTN by the year 2025 [2]. Importantly, three-quarters of the world's hypertensive population will reside in the low and middle income countries (LMICs) within the next decade [1].

In 2008, it was estimated that, globally, 4 in 10 adults over the age of 25 years and at the prime of their productivity were hypertensive [3]. Consequently, the burden is limited not only to the individual's health, but also to the significant economic burden and loss [2]. The WHO estimated that 3.76 trillion dollars will be an output lost and spent on cardiovascular diseases in the LMICs during the period 2011-2025 [2]. Hence, HTN is majorly affecting the nations' economic development of the LMICs due to the loss of income and the high costs of medical care [3].

The fact that the populations in the LMICs are bearing one of the highest burdens of the disease can be owed to the alarming rates of demographic changes including the growth and ageing of the populations, urbanization, and globalization [4]. These changes led to shifts in the lifestyle habits and behaviors, demonstrated mainly by the ongoing nutritional transition, the adoption of westernized high energy dense diets, and the reduced physical activity at the workplace and at leisure [4, 5].

The Arab world reported a higher crude prevalence of HTN (29.5%) when compared to other regions of the world such as the sub-Saharan Africa (27.6%) and the USA (28%) [6]. Lebanon, a small middle income Arab country, was reported to have higher prevalence rates of HTN when compared to its adjacent countries, such as Palestine and Egypt [7]. Findings of recent studies in Lebanon have showed that HTN affects one-third of the Lebanese population and an additional 30% are prehypertensive [7]. Of more concern is the observed increasing secular trend whereby Lebanon has witnessed a threefold increase in the prevalence of HTN during the past decade [4, 7, 8]. While the awareness and control rates of HTN in Lebanon (53%, 27%, respectively) are found to be better than the adjacent countries [7] yet these rates remain low compared to the high income ones [9].

Epidemiological data has revealed the need for increased awareness of HTN especially in low and middle income countries where the public awareness of the disease is moderately dismal [10, 11] as well as for abundant research on the management of the disease. The timely exploration of the burden of hypertension in the Lebanese community is fundamental, as it serves in guiding healthcare policy makers and public health providers to implement effective and tailored interventions for better management of the disease. Hence, this study aimed at (1) assessing the prevalence of HTN and pre-HTN amongst adults in Lebanon; (2) assessing the awareness and control rates of HTN; (3) investigating the factors associated with HTN, unaware HTN, and uncontrolled HTN in this population.

## 2. Methods

### 2.1. The Study Design and Target Population

This was a community based cross-sectional study conducted in 2014 in Greater Beirut Area (GBA). The study recruitment was done at the American University of Beirut (AUB) over a 3-month period from March until May 2014. The study included Lebanese adults aged 18-79 years and residing in GBA. It excluded vulnerable populations, mainly pregnant and lactating women, dialysis patients, and subjects with mental disabilities. The study was approved by the Institutional Review Board of AUB. A study by Nasrallah et al. reported the prevalence of type 2 diabetes in the Lebanese population from the collected data [12]. The study explained the methodology of the project elaborated below [12].

### 2.2. Sampling Strategy

The selection criteria were based on multistage probability sampling. First, the districts of Central Administrative Beirut in addition to areas in the districts of Chouf, Aley, Baabda, Metn, and Keserwan were selected as clusters. Second, within each selected cluster, neighborhoods were selected to represent the make-up of the areas, followed by the selection of the households which was based on a systematic random sampling according to the estimated number of buildings in the neighborhood. Finally, sampling a primary respondent within each household based on the most recent birthday was done. The objectives of the study along with the methods were clearly explained to the selected participants who accepted to get enrolled. Those who agreed on the objectives and conditions had signed an informed consent.

### 2.3. Data Collection

Information collected from subjects included (1) demographic and socioeconomic data: age, gender, marital status, education, and income level; (2) lifestyle-related data: smoking (current smoker defined as any daily smoking, regardless of the number of cigarettes or water-pipe), alcohol intake (defined as any intake), caffeine intake, and being physical active, assessed as (yes/no); (3) medical history: coronary artery disease and diabetes mellitus; (4) anthropometric measures: waist circumference and waist-to-hip ratio using a standardized method [13] and body composition using bioimpedance analyzer (Inbody Body Composition Analyzer, In body 230); sitting blood pressure and heart rate were obtained twice at 10-minute intervals using a digital sphygmomanometer; and (5) laboratory measures: fasting glucose, HbA_1C_, lipid profile, CRP, sodium urine, and potassium urine. (6) Dietary assessment was performed using an 80-item culture-specific semiquantitative food frequency questionnaire (QFFQ) that estimated food and beverage intakes over the past year [14]. The daily energy and macronutrient intake levels were computed using the food composition database of the Nutritionist Pro Software (Axxya Systems LCC 2016, Nutritionist Pro™ version 6.3.0. Stafford) [14, 15].

### 2.4. Outcome Related Variables

According to the thresholds of the Joint National Committee on Prevention, Detection, Evaluation and Treatment of High Blood Pressure Seven (JNC 7) and the (JNC 8) guidelines for the management of hypertension in adults, the participants were classified as follows:

- Hypertensive individuals: defined as those with SBP ≥140 mm Hg and/or DBP ≥ 90 mmHg [16] or those who have been informed of being diagnosed with HTN.
- Prehypertensive individuals: defined as those who have not been informed of HTN diagnosis and with SBP 120- 139 mm Hg and/or DBP 80-89 mm Hg [16] and not on pharmacologic treatment.
- Normotensive individuals: defined as those who have not been informed of HTN diagnosis and with SBP < 120 mm Hg and DBP < 80 mm Hg [16] and not on pharmacologic treatment.
- Aware individuals: defined as those who have been informed of HTN diagnosis [17].
- Unaware individuals: defined as those who have never been informed of HTN diagnosis and with SBP ≥140 mm Hg and/or DBP ≥ 90 mm Hg.
- Controlled HTN: defined as SBP < 140 mmHg for individuals below the age of 60; SBP < 150 mm Hg for individuals above the age of 60 years and DBP < 90 mmHg as a result of pharmacologic treatment among the aware hypertensive [18].
  - Treated individuals: defined as those who were aware of being hypertensive and are on pharmacologic treatment

### 2.5. Statistical Analysis

Descriptive statistics were conducted for the overall characteristics of the study population through presenting the numbers and percent for the categorical variables and means and standard deviations for the continuous ones. Inferential bivariate analysis was carried out where Chi square or Fisher exact tests were used for the categorical and binary factors, as appropriate. Independent t-test and one-way ANOVA tests were conducted for the continuous variables. Results were presented by the p-values in addition to the descriptive statistics for each of the outcome groups identified. Multiple and multinomial logistic regression were carried out to adjust for potential confounding and/or interaction effect of variables under study. The stepwise approach was used to choose the best model. The results were presented by the odds ratios and 95% confidence intervals (CI). P-value < 0.05 was set as an indicator of statistical significance. The data analysis was done on two types of software: SPSS 22 and STATA 13.

## 3. Results

A total of 501 subjects participated in the study. The sample consisted of 322 women (64.3%) and 179 men (35.7%), with a mean age of 45.4 ± 15.0 years. Approximately 10% of the study participants reported a monthly income above 2000 USD per household and university level of education. The lifestyle habits results showed that 43% of the participants were current cigarettes smokers, 28.3% were current nargileh smokers, and 19% were current alcohol drinkers. The majority of the study participants reported drinking coffee (80.4%) and engaging in physical activity (84.2%) (Table 1). The overall prevalence of HTN in GBA was 36.4%, 25.3% were pre-HTN, and the rest were normotensive (38.2%). The awareness rate among the hypertensive participants was 65.4% while the control rate amongst those who are on treatment and aware of being hypertensive was estimated at 61.2%.

### 3.1. Predictors of Pre-HTN and HTN

Table 2 presents the differences in the characteristics of the participants among the three BP groups (normotensive, pre-HTN, and HTN). The results showed that there was an increase in the mean age with the increase in BP categories, such that the mean age was 40.24 ± 12.8, 41.6 ± 14.2, and 53.6 ± 14.2 years for the normotensive, pre-HTN, and hypertensive, respectively (p-value <0.0001). Males were found to be more hypertensive (37.4%) than normotensive (25.1%) unlike women (p-value< 0.0001). Significant difference in the income level between the BP groups was detected as higher prevalence of HTN (51.2%) was observed among those who receive <600 USD compared to the normotensive (25.3%); this prevalence (15.0%) is lower among those who receive 1000-2000 USD compared to the normotensive (27.6%). Similar results were observed for the educational level.

BMI and abdominal obesity were found to be significant correlates. The percentage of the obese individuals increased significantly and gradually among the three groups (23.3% for the normotensive, 45.6% for the pre-HTN, and 59.3% for the hypertensive individuals). Furthermore, the results showed significant differences in the mean of the macronutrients (carbohydrates, total fat, and the saturated fats in addition to the total energy) among the different groups, where the highest mean of each of the mentioned macronutrients was among the pre-HTN group while the lowest was among the hypertensive group when compared to the normotensive (p-value <0.05) (Table 2).

Results of the logistic regression analyses showed that the main factors that were significantly associated with HTN were age, income level, T2D, triglyceride, and CRP (Table 3). Older age groups were at higher odds of having HTN in comparison to participants below the age of 30 years. The odds increased up to 3.53 (95% CI: 0.94-11.72) for the age group 51-60 years and to 17.91 (95% CI: 4.97- 64.45) for those above the age of 60 years. Interestingly, higher income levels were associated with lower odds of developing HTN, yet the only significant result was among those who earn more than 2000 USD per household with an OR = 0.22 (95% CI: 0.07-0.88). Besides, T2D was found to be a positive correlate which increases the odds of having HTN by 2.4 (95% CI: 1.08-7.02). Similarly, a positive association was obtained with the CRP and TG levels (OR = 1.46; 95% CI: 1.08-2.01) (OR = 1.04; 95% CI: 1.01-1.08). On the other hand, urinary potassium and HDL were the only predictors that were significantly associated with pre-HTN. Urinary potassium was a negative correlate of pre-HTN (p-value = 0.01), while HDL was found to be a positive one (p-value = 0.003).

The logistic regression analysis results (Table 3) showed that gender and body fat were the two common positive predictors for both the HTN and pre-HTN categories with almost equal strength of associations. The odds of being hypertensive (OR = 4.78; 95% CI: 2.25-11.11) and pre-HTN (OR = 3.71; 95% CI: 2.56-9.72) was higher among males compared to females. Similarly, for every unit increase in body fat, the odds of HTN and pre-HTN increased by 1.08 (95% CI: 1.05-1.12) and by 1.05 (95% CI: 1.02-1.09), respectively.

### 3.2. Predictors of Unaware HTN

Table 4 presents the differences in the characteristics between the normotensive participants and the unaware hypertensive. The bivariate results showed that unawareness was more common among older people, males, lower income level, obese, and those with higher levels of lipid profile. The mean age of the unaware hypertensive (47.6 ± 16.6 years) was significantly higher than that of the normotensive (40.2 ± 12.8 years) with a p-value = 0.002. Moreover, the income level was found to be significantly associated with the unawareness of the disease, with a p-value = 0.03. Interestingly, obesity was found to be more common among the unaware (58.7%) (p-value <0.0001) compared to the normotensive ones (23.0%) and with a higher mean of body fat (32.9 ± 12.3) than the normotensive (24.9 ± 9.7) (p-value = 0.001). Significant association at the bivariate level was also obtained between blood glucose, HbA1c, CRP, cholesterol, triglyceride, and LDL such that the mean of each mentioned lab test was higher among the unaware patients. Yet, the mean of the glomerular filtration rate was lower among the unaware patients (95.8 ± 21.1) than those who are normal (104.9 ± 22.8) (p-value = 0.006) which can be indicative of chronic kidney disease.

Upon adjustment, age was found to be the significant predictor with the strongest association for the unawareness. Significant results were reported among those who are above the age of 60 years, such that the odds for an older person to be unaware of being hypertensive increased up to OR = 7.36 as compared to those ≤ 30 years old (p-value = 0.01). Also, males were found to be at higher odds of being unaware of the disease with an OR = 5.15 (95% CI 2.16-12.25; p-value <0.0001) and the same applies to the single participants with an OR = 4.55 (95% CI: 1.16-17.76; p-value = 0.02). Higher BMI was more common among the unaware patients such that the odds of being unaware hypertensive patient among the obese were found to be 7 times more likely when compared to those with normal weight (OR = 6.83, 95% CI: 2.59-22.01; p<0.0001) (Table 5).

### 3.3. Predictors of Uncontrolled HTN

Table 6 presents the differences in the characteristics of the controlled hypertensive patients and the uncontrolled ones. The results showed that hypertensive males were more likely to be uncontrolled (37.5%). Similarly, obese participants were more uncontrolled (70.0%) compared to the controlled (79.4%). Yet, none of the studied factors showed a significant difference at the bivariate level. After adjustment, none of the predictors was found to be statistically significant in the final model.

## 4. Discussion

This cross-sectional study provided an estimate of the current prevalence and control rates of HTN in a community sample representative of the GBA adult population. It highlighted the burden of the disease: 36.4% of the study participants were hypertensive, 25.3% were prehypertensive, and only 38.2% had optimal BP. The awareness rate among the hypertensive participants was estimated at 65.4% and the control rate at 61%.

Our findings of HTN prevalence is comparable to those of a cross sectional study conducted in 2013 in all six provinces of Lebanon and including a sample of 1697 participants, which reported a crude prevalence of 36.9% for HTN and 30% for pre-HTN while the control rate was 54% [7]. On the other hand, prevalence of the pre-HTN group is lower in GBA compared to the national level [7]. Both studies utilized similar methodologies, specifically the definitions of BP, which was based on BP measurements and not on self-report only. Interestingly, the control rate in GBA remained higher when compared to the national study. This can be possibly justified by the effect of urban living of our study setting. A study showed that low rates of treatment and management of HTN were obtained in the rural areas of the low to middle income countries, which was mainly due to difficulties in the accessibility to healthcare [19]. Population living in the urban settings does not encounter the same factors in terms of accessibility as those who are in rural areas, such as the costs in accessing healthcare centers, the distance to clinics, and the difference in the quality of care provided [19].

Comparing the findings of our study with similar studies in the adjacent countries, Lebanon had the higher prevalence of HTN when compared to Palestine (27.6%), Egypt (26.3%), and Turkey (31.8%) [20–22]. What ameliorates this finding is that Lebanon had better control rates of the disease compared to the rates reported from Palestine (9.5%), Egypt (8%), and Turkey (8.1%), respectively [20–22]. On the other hand, comparing the results with the West, Lebanon had higher prevalence of HTN than the developed countries such as the USA (29%) [23] and Canada (20%) [24]. Yet the control rate in Lebanon is comparable to the same countries, USA (63%) and Canada (66%) [9]. The variability across countries is multifactorial and could have occurred because of differences in the study designs and methodologies, time frames, geographic variations, lifestyle habits, and socioeconomic differences in addition to medical access and quality of care [6].

Regression analyses showed that increasing age, male gender and T2D were positive correlates for HTN. The findings were in concordance to the results of the national study by Matar et al. with similar strengths of associations [7]. In contrast to Matar et al. findings, our study identified income level as a significant correlate for HTN. The reported results showed that subjects with higher income level had lower prevalence of HTN. Our results are consistent with those reported in other studies [25, 26]. A study conducted in the United Arab Emirates demonstrated that HTN was found to be significantly higher among low income groups [25]. Another study conducted in Canada reported that income was also a negative independent correlate for HTN [26]. Therefore, several studies have found that income is a crucial socioeconomic measure to examine variables that affect the health, as it provides access to other factors such as education, medical care, goods, and services [25, 26]. A lower income level and the challenging life conditions can justify the unhealthy lifestyle habits which could influence behaviors, leading to a higher risk of HTN [27]. Moreover, previous evidence demonstrated that socioeconomic status (SES) including the income level can shape and direct the lifestyle habits and behaviors of individuals [28]. Accordingly, maybe more effort to screen for HTN among individuals of lower income level ought to be directed.

Additionally, body fat was found to be high among the hypertensive and prehypertensive population of GBA when compared to the normal. Likewise, TG and CRP are biochemical factors that were positively associated with HTN. High TG, body fat, and CRP are factors linked to the metabolic syndrome which increases the overall cardiovascular risk [29, 30].

On the other hand, potassium was found to be the only dietary factor that is significantly negatively associated with pre-HTN. A study reported that, in borderline hypertensive patients, a low-potassium diet (16 mmol/day) for 10 days increases systolic and diastolic pressures by 7 and 6 mmHg, respectively, relative to 10 days on a high-potassium diet (96 mmol/day) [31]. Therefore, the adequate dietary intake of potassium can have an antihypertensive effect. The Dietary Approaches to Stop HTN (DASH) which relies mainly on increased consumption of fruits and vegetables, which are high in potassium, is a possible recommendation for the prehypertensive group [32]. None of the macronutrients was found to be significantly correlated to HTN and pre-HTN after the adjustment. Nevertheless, the dietary intakes from fats, carbohydrates, proteins, sodium, and total calories were found to be lower among the hypertensive when compared with the normal. These findings suggest that patients are possibly following modifications in their dietary habits for better control and management of the disease. Yet, none of the results were statistically significant.

Unaware hypertensive patients among community members not known to have HTN were mostly above the age of 60, males, single, and obese. Interestingly, the SES was no longer significant after the adjustment. Comparing the findings with those of Matar et al. (2015), results were similar showing that HTN awareness was poorer in males when compared to females and in single individuals compared to the married, yet our findings showed that unawareness was among the older subjects and those who had diabetes or hyperlipidemia [7]. Results from adjacent countries showed higher hypertension awareness is among women, older hypertensive, diabetics, obese, housekeepers, and those who have high physical activity levels [11, 33]. Similarly, male sex and older age were the main factors associated with unawareness of hypertension in a study done by Hyman and Pavlik in the United States [34].

Healthcare access and utilization play a major role in increasing the awareness of HTN. Studies showed that gender difference in the healthcare use is one of the main reasons contributing to the differences in the awareness of the disease [35–37]. Women are more likely to seek care from health practitioners, especially for gynecological services; on the other hand, heteronormative masculinity scripts dictate men to be tough and not seek help in times of need [35, 36].

We could not identify any predictor of HTN control in treated aware hypertensive patients. The control of HTN relies on the modifications in the lifestyle habits and on pharmacologic treatment [2]. Our findings could not detect any significant association for dietary and behavioural habits. It is important to note that the dietary assessment performed in this study was based on a food frequency questionnaire, which may be limited by measurement errors, reliance on memory, and the number of food items included in the food list [38]. However, despite these potential limitations, the FFQ was shown to be the most suitable dietary assessment tool in large epidemiological studies since it assesses the subject's habitual diet over longer periods of time [39]. Other factors not studied might contribute to a poor control of HTN such as medication adherence, adequate pharmacologic treatment, psychological stress, access to healthcare, and patients' knowledge of the target BP level. A Lebanese national study that attempted to identify predictors of BP control reported that diabetes was a poor predictor for BP control, whereas the early control and the combination therapy were for better control [40]. Another national Lebanese study by Farah et al. aimed to assess the factors contributing to the control of HTN [41]. The major factors that were found to be correlated with uncontrolled BP were the low medication adherence and obesity [41]. The findings of the literature are still not enough and BP control remains a major public health challenge in Lebanon.

### 4.1. Limitations and Strengths

The study has several limitations. Being a cross-sectional study, general associations and hypothesis may be derived, but temporal relationship and causality cannot be established. Even though it is a community based study, selection bias is another limitation due to the small cohort of participants enrolled in the study and the female overrepresentations. The national statistics show that one-third of the Lebanese population are residing in GBA where 50.6% of the population are females while 49.4% are males [7]. The high female to male ratio in our study can be due to data collection that was done during the week days and working hours. It is possible that those who are unemployed and housewives were more likely to participate. Also, the sample was taken only from GBA which limits the generalizability of the results to the whole country. On the other hand, GBA is a major part of Lebanon where the national statistics report that 47.7% of the Lebanese population are residing in this area [7]. Therefore, the findings of this study could be considered representative for urban adults in Lebanon, which provides a ground for further epidemiologic investigations and comparison. Furthermore, variables in the study relied on biochemical and anthropometric measures rather than personal reports from subjects, hence giving us accurate and reliable data foundation to build our conclusions on.

## 5. Conclusion

Our findings showed that the prevalence of HTN is consistently high, yet there is an improvement in the awareness and management of the disease. The identified predictors of HTN in GBA were the same as those presented in previous studies done in Lebanon. However, income level, body fat, and CRP were additional factors identified among HTN patients in GBA. Interestingly, among the unaware hypertensive patients who perceive themselves as normal, obesity remains a major problem in the population. Furthermore, our study could not identify any predictor for HTN control and further investigations are needed.

Our results can advise the development and establishment of national interventions by the public health sectors to achieve better awareness, primary prevention, and better control of the disease. The development of a national awareness campaign for hypertension can serve in increasing the detection of the disease, educating the community on factors impacting their BP level, and promoting the importance of following healthy lifestyle habits (healthy diet) and medication adherence.

## Figures and Tables

**Table 1:** Baseline characteristics of the study sample.

			**N (**%**)**
**Total sample **			501

**Demographic**	**Age**	≤ 30	107 (21.4%)
		31-40	78 (15.6%)
		41-50	118 (23.6%)
		51-60	123 (24.6%)
		> 60	75 (14.9%)
	**Age**	mean ± SD	45.4 ± 15.0
	**Gender**	Males	179 (35.7%)
		Females	322 (64.3%)
	**Marital status**	Married	332 (66.3%)
		Single	98 (19.6%)
		Others	71 (14.2%)

**Socioeconomic**	**Income**	<600$	153 (33.8%)
		600-999.9$	170 (37.5%)
		1000-2000$	90 (19.9%)
		>2000$	40 (8.8%)
	**Education**	No school /Primary	181 (36.3%)
		Intermediate/Secondary/Technical	263 (52.8%)
		University degree	54 (10.8%)

**Lifestyle habits**	**Smoking cigarettes**	Never	236 (47.1%)
		Current	216 (43.1%)
		Ex-smoker	49 (9.8%)
	**Narghileh Smoking**	Never	311 (62.1%)
		Current	142 (28.3%)
		Ex-smoker	48 (9.6%)
	**Alcohol drinking**	Never	372 (74.3%)
		Current	95 (19.0%)
		Ex-smoker	34 (6.8%)
	**Coffee drinking**		403 (80.4%)
	**Physical activity**		422 (84.2%)

**Table 2:** The association of demographic, socioeconomic, lifestyle, anthropometric, dietary intake, and medical history and laboratory tests with BP.

**Variables**			**Normotensive**	**Pre-HTN**	**HTN**	**p-value**
			**N = 191**	**N = 127**	**N = 182**	
			**38.2**%	**25.3%**	**36.4**%	
**Demographic**	**Age**	**Mean (± sd)**	40.2 ± 12.8	41.6 ± 14.2	53.6 ± 14.2	<0.0001^*∗*^
	**Age**	**≤ 30**	51 (26.7%)	36 (28.3%)	19 (10.4%)	<0.0001^*∗*^
		**31-40**	42 (22.0%)	23 (18.1%)	13 (7.1%)	
		**41-50**	57 (29.8%)	32 (25.2%)	29 (15.9%)	
		**51-60**	34 (17.8%)	23 (18.1%)	66 (36.3%)	
		**> 60**	7 (4.0%)	13 (10.2%)	55 (30.2%)	
	**Gender**	**Females**	143 (74.9%)	64 (50.4%)	114 (62.6%)	<0.0001^*∗*^
		**Males**	48 (25.1%)	63 (49.6%)	68 (37.4%)	
	**Marital status**	**Married**	138 (72.3%)	80 (63.0%)	113 (62.1%)	<0.0001^*∗*^
		**Single**	39 (20.4%)	35 (27.6%)	24 (13.2%)	
		**Others**	14 (7.3%)	12 (9.4%)	45 (24.7%)	

**Socioeconomic**	**Income**	**<600$**	44 (25.3%)	27 (22.9%)	82 (51.2%)	<0.0001^*∗*^
		**600-999.9$**	64 (36.8%)	58 (49.2%)	47 (29.4%)	
		**1000-2000$**	48 (27.6%)	18 (15.3%)	24 (15.0%)	
		**>2000$**	18 (10.3%)	15 (12.7%)	7 (4.4%)	
	**Educational**	**No school /Primary**	50 (26.6%)	44 (34.6%)	87 (47.8%)	<0.0001^*∗*^
		**Intermediate/Secondary/Technical**	114 (60.6%)	65 (51.2%)	83 (45.6%)	
		**University degree**	24 (12.8%)	18 (14.2%)	12 (6.6%)	

**Lifestyle habits**	**Cigarette smoking**	**Never**	90 (47.1%)	59 (46.5%)	86 (47.3%)	0.09
		**Current**	89 (46.6%)	57 (44.9%)	70 (38.5%)	
		**Ex-smoker**	12 (6.3%)	11 (8.7%)	26 (14.3%)	
	**Narghileh smoking**	**Never**	108 (56.5%)	78 (61.4%)	124 (68.1%)	0.11
		**Current**	62 (32.5%)	40 (31.5%)	40 (22.0%)	
		**Ex-smoker**	21 (11.0%)	9 (7.1%)	18 (9.9%)	
	**Coffee drinking**	**No**	41 (21.5%)	29 (22.8%)	27 (14.8%)	0.14
		**Yes**	150 (78.5%)	98 (77.2%)	155 (85.2%)	
	**Alcohol drinking**	**Never**	151 (79.1%)	85 (66.9%)	135 (74.2%)	0.08
		**Current**	31 (16.2%)	33 (26.0%)	31 (17.0%)	
		**Ex-drinker**	9 (4.7%)	9 (7.1 %)	16 (8.8%)	
	**Physical activity**	**None**	27 (14.1%)	21 (16.5%)	31 (17.0%)	0.71
		**Any activity**	164 (85.9%)	106 (83.5%)	151 (83.0%)	

**Anthropometric measurements**	**BMI**	**Normal**	66 (34.9%)	34 (27.2%)	16 (8.8%)	<0.0001^*∗*^
		**Overweight**	79 (41.8%)	34 (27.2%)	58 (31.9%)	
		**Obese**	44 (23.3%)	57 (45.6%)	108 (59.3%)	
	**Abdominal obesity –waist circumference**		83 (43.7%)	59 (46.8%)	136 (74.7%)	<0.0001^*∗*^
	**Abdominal obesity- waist to hip ratio**		152 (80.4%)	82 (65.6%)	149 (82.3%)	0.001^*∗*^
	**Body fat**	**Mean (± sd)**	24.9 ± 9.8	27.5 ± 12.3	33.4 ± 11.1	<0.0001^*∗*^

**Dietary intake**	**Total energy –Kcal**	**Mean (± sd)**	3291.0 ± 1532.3	3714.2 ± 1534.5	3057.4 ± 1473.2	0.01^*∗*^
	**Total protein (g/d)**	**Mean (± sd)**	108.9 ± 77.2	118.7 ± 54.2	101.9 ± 60.1	0.14
	**Carbohydrates (g/d)**	**Mean (± sd)**	390.5 ± 224.2	438.8 ± 268.7	374.9 ± 211.8	0.05^*∗*^
	**Total fat (g/d)**	**Mean (± sd)**	144.4 ± 89.4	159.3 ± 96.1	129.7 ± 78.1	0.01^*∗*^
	**Saturated fat (g/d)**	**Mean (± sd)**	38.5 ± 25.4	43.65 ± 31.8	32.9 ± 21.3	0.002^*∗*^
	**% of calories from carbohydrates**	**Mean (± sd)**	48.8 ± 8.7	48.5 ± 7.8	49.5 ± 8.7	0.57
	%** of calories from proteins**	**Mean (± sd)**	13.2 ± 3.9	12.9 ± 2.6	13.5 ± 3.0	0.40
	%** of calories from fat**	**Mean (± sd)**	38.9 ± 7.8	38.9 ± 7.6	37.9 ± 8.7	0.28
	%** of calories from saturated fat**	**Mean (± sd)**	10.2 ± 2.7	10.2 ± 2.8	9.5 ± 2.7	0.03^*∗*^

**Medical History**	**CAD**	**Cardiac catheterization**	10 (5.2%)	6 (4.7%)	29 (15.9%)	<0.0001^*∗*^
		**Previous heart attack**	7 (3.7%)	2 (1.6%)	13 (7.1%)	0.05^*∗*^
		**Family history**	70 (36.6%)	56 (44.1%)	63 (34.6%)	0.22
	**T2D**		9 (4.7%)	12 (9.4%)	54 (29.7%)	<0.0001^*∗*^

**Lab tests**	**Glucose**	**Mean (± sd)**	100.3 ± 28.0	107.5 ± 32.7	127.1 ± 56.6	<0.0001^*∗*^
	**HbA1c**	**Mean (± sd)**	5.5 ± 0.8	5.8 ± 1.1	6.4 ± 1.7	<0.0001^*∗*^
	**CRP**	**Mean (± sd)**	10.2 ± 7.2	11.3 ± 8.2	14.76 ± 13.0	<0.0001^*∗*^
	**Cholesterol**	**Mean (± sd)**	182.2± 37.5	182.8 ± 40.3	192.3 ± 50.1	0.052
	**TG**	**Mean (± sd)**	118.8 ± 68.8	143.3 ± 145.9	164.3 ± 88.4	<0.0001^*∗*^
	**HDL**	**Mean (± sd)**	50.8 ± 14.8	51.5 ± 16.4	46.9 ± 13.1	0.01^*∗*^
	**LDL**	**Mean (± sd)**	107.0 ± 32.9	105.1 ± 35.4	113.3 ± 48.3	0.12
	**Sodium urine**	**Mean (± sd)**	126.2 ± 47.9	125.6 ± 57.4	113.7 ± 48.3	0.03^*∗*^
	**Potassium urine**	**Mean (± sd)**	79.3 ± 34.9	69.6 ± 31.8	76.2 ± 32.9	0.03^*∗*^
	**GFR (Ml/min/1.73 m** ^**2**^ **)**	**Mean (± sd)**	104.9 ± 22.8	102.3 ± 23.9	93.0 ± 26.1	<0.0001^*∗*^

**Table 3:** The multinomial logistic regression model for the BP groups.

		**Normotensive**	**Pre-HTN**	**OR (95**%** CI)**	**HTN**	**OR (95**%** CI)**
				**Pre-HTN/Normotensive**		**HTN/Normotensive**
**Age**	**≤ 30**	51 (26.7%)	36 (28.3%)	Ref	19 (10.4%)	Ref
	**31-40**	42 (22.0%)	23 (18.1%)	1.04 (0.43-2.59)	13 (7.1%)	1.39 (0.46-4.19)
	**41-50**	57 (29.8%)	32 (25.2%)	1.25 (0.50-3.02)	29 (15.9%)	1.72 (0.57-5.19)
	**51-60**	34 (17.8%)	23 (18.1%)	1.39 (0.43-3.04)	66 (36.3%)	4.93 (1.67-14.51)
	**≥ 60**	7 (3.7%)	13 (10.2%)	3.53 (0.94-11.72)	55 (30.2%)	17.91 (4.97- 64.45)

**Gender**	**Females**	143 (74.9%)	64 (50.4%)	Ref	114 (62.6%)	Ref
	**Males**	48 (25.1%)	63 (49.6%)	3.71 (2.56-9.72)	68 (37.4%)	4.78 (2.25-11.11)

**Marital Status**	**Married**	138 (72.3%)	80 (63.0%)	Ref	113 (62.1%)	Ref
	**Single**	39 (20.4%)	35 (27.6%)	1.82 (0.71-4.41)	24 (13.2%)	3.57 (1.02-8.09)
	**Others**	14 (7.3%)	12 (9.4%)	1.15 (0.43-2.96)	45 (24.7%)	2.22 (0.95-5.16)

**Income level**	**<600 $**	44 (25.3%)	27 (22.9%)	Ref	82 (51.2%)	Ref
	**600-999.9$**	64 (36.8%)	58 (49.2%)	1.56 (0.83-3.14)	47 (29.4%)	0.67 (0.38-1.42)
	**1000-2000$**	48 (27.6%)	18 (15.3%)	0.64 (0.29-1.44)	24 (15.0%)	0.55 (0.25-1.19)
	**>2000$**	18 (10.3%)	15 (12.7%)	1.03 (0.38-2.98)	7 (4.4%)	0.22 (0.07-0.88)

**T2D**		9 (4.7%)	12 (9.4%)	1.21 (0.44-3.87)	54 (29.7%)	2.41 (1.08-7.02)

**Abdominal obesity ** **(waist to hip ratio)**		152 (80.4%)	82 (65.6%)	0.54 (0.21-1.43)	149 (82.3%)	1.09 (0.41-2.89)

**Body fat **	**Mean (sd)**	24.9 ± 9.8	27.5 ± 12.3	1.05 (1.02-1.09)	33.4 ± 11.1	1.08 (1.05-1.12)

**Urinary potassium (10 units)**	**Mean (sd)**	79.3 ± 34.9	69.6 ± 31.8	0.89 (0.98-0.99)	76.2 ± 32.9	0.95 (0.98-1.003)

**TG (10 units)**	**Mean (sd)**	118.8 ± 68.8	143.3 ± 145.9	1.01 (0.99-1.01)	164.3 ± 88.4	1.04 (1.01-1.08)

**HDL (10units)**	**Mean (sd)**	50.8 ± 14.8	51.5 ± 16.4	1.37 (1.01-1.05)	46.9 ± 13.1	1.15 (0.93-1.42)

**CRP (10 units)**	**Mean (sd)**	10.2 ± 7.2	11.3 ± 8.2	1.17 (0.82-1.62)	14.76 ± 13.0	1.46 (1.08-2.01)

**Table 4:** The association of demographic, socioeconomic, lifestyle, anthropometric, dietary intake, and medical history and laboratory tests with unaware hypertensive.

**Variables**			**Normotensive**	**Unaware HTN**	**P-value**
**N = 254**			**N = 191**	**N = 63**	
			**(75.2**%**)**	**(24.8**%**)**	
**Demographics**	**Age**	**Mean (± sd)**	40.2 ± 12.8	47.6 ± 16.6	0.002
	**Age**	**≤ 30**	51 (26.7%)	16 (25.4%)	0.002
		**31-40**	42 (22.0%)	5 (7.9%)	
		**41-50**	57 (29.8%)	8 (12.7%)	
		**51-60**	34 (17.8%)	20 (31.7%)	
		**≥ 60**	7 (3.7%)	14 (22.2%)	
	**Gender**	**Males**	48 (25.1%)	34 (54.0%)	<0.0001
		**Females**	143 (74.9%)	29 (46.0%)	
	**Marital Status**	**Married**	138 (72.3%)	32 (50.8%)	0.002
		**Single**	39 (20.4%)	18 (28.6%)	
		**Others**	14 (7.3%)	13 (20.6%)	

**Socioeconomic**	**Income Level**	**<600 $**	44 (25.3%)	25 (44.6%)	0.03
		**600-999.9$**	64 (36.8%)	16 (28.6%)	
		**1000-2000$**	48 (27.6%)	9 (16.1%)	
		**>2000$**	18 (10.3%)	6 (10.7%)	
	**Education Level**	**No school/Primary**	50 (26.6%)	23 (36.5%)	0.33
		**Intermediate/Secondary/Technical**	114 (60.6%)	33 (52.4%)	
		**University degree**	24 (12.8%)	7 (11.1%)	

**Lifestyle habits**	**Cigarette smoking**	**Never**	90 (47.1%)	28 (44.4%)	0.025
		**Current**	89 (46.6%)	24 (38.1%)	
		**Ex-smoker**	12 (6.3%)	11 (17.5%)	
	**Narghileh Smoking**	**Never**	108 (56.5%)	37 (58.7%)	0.63
		**Current**	62 (32.5%)	17 (27.0%)	
		**Ex-smoker**	21 (11.0%)	9 (14.3%)	
	**Coffee drinking**	**No**	41 (21.5%)	12 (19.0%)	0.68
		**Yes**	150 (78.5%)	51 (81.0%)	
	**Alcohol drinking**	**Never**	151 (79.1%)	45 (71.4%)	0.41
		**Current**	31 (16.2%)	13 (20.6%)	
		**Ex-drinker**	9 (4.7%)	5 (7.9%)	
	**Physical Activity**	**None**	27 (14.1%)	9 (14.3%)	0.97
		**Yes**	164 (85.9%)	54 (85.7%)	

**Anthropometric Measurements**	**BMI**	**Normal**	66 (34.6%)	10 (15.9%)	<0.0001
		**Overweight**	79 (41.4%)	16 (25.4%)	
		**Obese**	44 (23.0%)	37 (58.7%)	
	**Waist Circumference- Abdominal Obesity**	**No**	107 (56.3%)	23 (36.5%)	0.005
		**Yes**	83 (43.7%)	40 (63.5%)	
	**Waist to hip ratio- Abdominal Obesity**	**No**	37 (19.6%)	13 (21.0%)	0.82
		**Yes**	152 (80.4%)	49 (79.0 %)	
	**Body Fat**	**Mean (± sd)**	24.9 ± 9.7	32.9 ± 12.3	0.001

**Dietary intake**	**Total Energy -Kcal**	**Mean (± sd)**	3134.0 ± 1532.3	3268.6 ± 1487.6	0.55
	**Total Protein (g/d)**	**Mean (± sd)**	104.7 ± 71.2	107.7 ± 55.8	0.76
	**Carbohydrates**	**Mean (± sd)**	372.5 ± 174.1	396.9 ± 175.2	0.34
	**Total Fat**	**Mean (± sd)**	137.4 ± 76.5	139.7 ± 70.62	0.84
	**Saturated Fat**	**Mean (± sd)**	36.7 ± 22.4	37.7 ± 20.4	0.76
	%** of calories from carbs**	**Mean (± sd)**	48.8 ± 8.7	48.9 ± 7.6	0.88
	%** of calories from proteins**	**Mean (± sd)**	13.2 ± 3.9	13.3 ± 2.9	0.73
	%** of calories from fat**	**Mean (± sd)**	38.9 ± 7.9	38.3 ± 7.4	0.56
	%** of calories from saturated fat**	**Mean (± sd)**	10.2 ± 2.7	10.3 ± 3.0	0.72

**Medical History**	**CAD**	**Cardiac catheter**	10 (5.2%)	2 (3.2%)	0.73
		**Previous heart attack**	7 (3.7%)	2 (3.2%)	1.00
		**Family history**	70 (36.6%)	24 (38.1%)	0.83
	**T2D**		9 (4.7%)	6 (9.5%)	0.21

**Lab tests**	**Glucose**	**Mean (± sd)**	100.3 ± 28.1	112.1 ± 34.5	0.01
	**HbA1c**	**Mean (± sd)**	5.5 ± 0.8	5.9 ± 1.3	0.01
	**CRP**	**Mean (± sd)**	10.1 ± 7.1	13.9 ± 9.8	0.006
	**Cholesterol**	**Mean (± sd)**	182.2 ± 37.5	198.4 ± 53.1	0.008
	**TG**	**Mean (± sd)**	118.7 ± 68.8	153.8 ± 87.2	0.001
	**HDL**	**Mean (± sd)**	50.8 ± 14.8	47.7 ± 13.1	0.13
	**LDL**	**Mean (± sd)**	107.0 ± 32.8	125.5 ± 49.0	0.028
	**Sodium urine**	**Mean (± sd)**	126.2 ± 47.9	125.5 ± 49.0	0.92
	**Potassium urine**	**Mean (± sd)**	79.3 ± 34.8	76.3 ± 35.6	0.54

	**GFR**	**Mean (± sd)**	104.9 ± 22.8	95.8 ± 21.1	0.006

**Table 5:** Multiple logistic regression model for the unaware hypertensive versus the normotensive participants.

		**Odds Ratio**	**95 **%** CI**	**P-value**
**Age **	**≤ 30**	**Ref**	**Ref**	**Ref**
	**31-40**	1.09	0.25-4.65	0.91
	**41-50**	1.90	0.41-8.78	0.41
	**51-60**	3.39	0.81-14.21	0.09
	**>60**	7.36	1.18-33.07	0.01

**Gender**	**Females**	**Ref**	**Ref**	**Ref**
	**Males**	4.57	1.97-10.59	<0.0001

**Marital Status **	**Married**	**Ref**	**Ref**	**Ref**
	**Single**	4.55	1.16-17.76	0.02
	**Others**	2.27	0.68-7.54	0.17

**Income Level**	**<600 $**	**Ref**	**Ref**	**Ref**
	**600-999.9$**	0.69	0.27-1.78	0.20
	**1000-2000$**	0.51	0.17-1.53	0.18
	**>2000$**	0.26	0.06-1.15	0.07

**BMI **	**Normal**	**Ref**	**Ref**	**Ref**
	**Overweight**	2.53	0.83-7.69	0.16
	**Obese**	6.83	2.59-22.01	<0.0001

**Table 6:** The association of demographic, socioeconomic, lifestyle, anthropometric, dietary intake, and medical history and laboratory test with the uncontrolled HTN.

**Variables**			**Controlled HTN**	**Uncontrolled HTN**	**P-value**
**N = 103**			**N = 63 (61.2**%**)**	**N = 40 (38.8**%**)**	
**Demographics**	**Age**	**≤ 30**	0(0%)	0 (0%)	0.75
		**31-40**	2(3.2%)	2 (5.0%)	
		**41-50**	12 (19.0%)	5 (12.5%)	
		**51-60**	25 (39.7%)	16 (40.0%)	
		**≥ 60**	24 (38.1%)	17 (42.5%)	
	**Gender**	**Males**	14 (22.2%)	15 (37.5%)	0.09
		**Females**	49 (77.8%)	25 (62.5%)	
	**Marital status**	**Married**	43 (68.3%)	29 (72.5%)	0.83
		**Single**	1 (1.6%)	1 (2.5%)	
		**Others**	19 (30.2%)	10 (25.0%)	

**Socioeconomics**	**Income**	**<600 $**	33 (61.1%)	20 (55.6%)	0.74
		**600-999.9$**	15 (27.8%)	11 (30.6%)	
		**1000-2000$**	6 (11.1%)	4 (11.1%)	
		**>2000$**	0 (0.0%)	1 (2.8%)	
	**Education**	**No school /Primary**	30 (61.2%)	14 (56.0%)	0.37
		**Intermediate/Secondary/Technical**	18 (36.7%)	10 (40.0%)	
		**University degree**	1 (2.0%)	1 (4.0%)	

**Lifestyle habits**	**Cigarette smoking**	**Never**	31 (49.2%)	20 (50.0%)	0.44
		**Current**	22 (34.9%)	17 (42.5%)	
		**Ex-smoker**	10 (15.9%)	3 (7.5%)	
	**Narghileh smoking**	**Never**	46 (73.0%)	32 (80.0%)	0.34
		**Current**	13 (20.6%)	4 (10.0%)	
		**Ex-smoker**	4 (6.3%)	4 (10.0%)	
	**Coffee drinking**	**No**	6 (9.5%)	7 (17.5%)	0.36
		**Yes**	57 (90.5 %)	33 (82.5%)	
	**Alcohol drinking**	**Never**	53 (84.1%)	27 (67.5%)	0.12
		**Current**	7 (11.1%)	8 (20.0%)	
		**Ex-drinker**	3 (4.8%)	5 (12.5%)	
	**Physical activity**	**None**	9 (14.3%)	11 (27.5%)	0.13
		**Yes**	54 (85.7%)	29 (72.5%)	

**Anthropometric measurements**	**BMI (Kg/m** ^**2**^ **)**	**Normal**	3 (4.8%)	2 (5.0%)	0.33
		**Overweight**	24 (38.1%)	10 (25.0%)	
		**Obese**	36 (57.1%)	28 (70.0%)	
	**Waist circumference- abdominal obesity**	**No**	13 (20.6%)	5 (12.5%)	0.42
		**Yes**	50 (79.4%)	35 (87.5%)	
	**Waist to hip ratio- abdominal obesity**	**No**	7 (11.1%)	6 (15.0%)	0.76
		**Yes**	56 (88.9%)	34 (85.0%)	
	**Body fat**	**Mean (± sd)**	34.6 ± 9.9	37.2 ± 8.4	0.27

**Dietary intake**	**Total energy -Kcal**	**Mean (± sd)**	2795.7 ± 1439.0	2638.4 ± 1307.8	0.58
	**Total protein (g/d)**	**Mean (± sd)**	96.6 ± 57.7	87.1 ± 44.2	0.38
	**Carbohydrates**	**Mean (± sd)**	348.7 ± 188.8	336.0 ± 221.9	0.75
	**Total fat**	**Mean (± sd)**	115.9 ± 68.9	107.3 ± 61.6	0.52
	**Saturated fat**	**Mean (± sd)**	27.7 ± 15.5	24.7 ± 13.2	0.32
	%** from carbs**	**Mean (± sd)**	50.3 ± 9.4	50.5 ± 9.6	0.93
	%** from proteins**	**Mean (± sd)**	13.7 ± 3.0	13.7 ± 3.4	0.95
	%** from fat**	**Mean (± sd)**	36.9 ± 9.1	36.7 ± 10.4	0.89
	%** from saturated fat**	**Mean (± sd)**	8.9 ± 2.4	8.5 ± 2.5	0.42

**Medical History**	**CAD**	**Cardiac catheter**	14 (22.2%)	11 (27.5%)	0.54
		**Previous heart attack**	6 (9.5%)	6 (15.0%)	0.53
		**Family history**	20 (31.7%)	14 (35.0%)	0.73
	**T2D**		27 (42.9%)	18 (45.0%)	0.83

**Lab tests**	**Glucose**	**Mean (± sd)**	131.5 ± 62.6	132.26 ± 62.6	0.13
	**HbA1c**	**Mean (± sd)**	6.5 ± 1.6	7.1 ± 2.0	0.13
	**CRP**	**Mean (± sd)**	14.1 ± 9.2	17.4 ± 19.0	0.24
	**Cholesterol**	**Mean (± sd)**	186.5 ± 42.3	196.1 ± 57.1	0.33
	**TG**	**Mean (± sd)**	160.1 ± 78.2	192.4 ± 95.7	0.06
	**HDL**	**Mean (± sd)**	47.3 ± 13.0	45.3 ± 11.7	0.43
	**LDL**	**Mean (± sd)**	107.3 ± 37.0	112.4 ± 46.6	0.53
	**Sodium urine**	**Mean (± sd)**	101.7 ± 48.9	112.2 ± 39.8	0.26
	**Potassium urine**	**Mean (± sd)**	74.4 ± 31.2	76.5 ± 35.2	0.74
	**GFR**	**Mean (± sd)**	90.1 ± 26.2	83.6 ± 27.9	0.23

## Data Availability

The data used to support the findings of this study are available from the corresponding author upon “reasonable” request.

## Abbreviations

BP: Blood pressure
BMI: Body mass index
CVD: Cardiovascular diseases
CKD: Chronic kidney diseases
FBS: Fasting blood sugar
GBA: Greater Beirut Area
GFR: Glomerular filtration rate
HDL: High density lipoprotein
HbA1c: Glycosylated hemoglobin
HTN: Hypertension
JNC7: Joint National Committee 7
JNC8: Joint National Committee 8
Pre-HTN: Prehypertension
LDL: Low density lipoprotein
LMICs: Low and middle income countries
Mg: Milligram
Mg/dl: Milligram per deciliter
MmHg: Millimeter of mercury
OR: Odds ratio
SES: Socioeconomic status
SBP: Systolic blood pressure
TG: Triglyceride
T2D: Type 2 diabetes.
